# Khat chewing and cirrhosis in Somaliland: Case series

**DOI:** 10.4102/phcfm.v8i1.1124

**Published:** 2016-07-28

**Authors:** Hawa D. Mahamoud, Sabah Mohammed Muse, Lewis R. Roberts, Philip R. Fischer, Michael S. Torbenson, Tim Fader

**Affiliations:** 1 Department of Family Medicine, Amoud University, Somaliland; 2 Mayo Clinic, Minnesota, United States

## Abstract

**Background:**

Khat chewing is common especially among men in East Africa and Yemen. It is generally viewed by the populace as a benign social custom. Several studies of ethnic Somali immigrants to Western countries suggest an association between khat chewing and hepatotoxicity, but the risk of hepatotoxicity related to khat chewing within African settings is not documented.

**Aim:**

To identify and describe liver disease without evidence of alcohol exposure or infectious etiology in khat chewers.

**Settings:**

A university-affiliated teaching hospital in Somaliland.

**Methods:**

Cases of cirrhosis of unknown cause were identified from the clinical practice of Al Hayatt Hospital in Borama, Somaliland, during 14 months beginning December 2012.

**Results:**

Eight Somali men aged 27–70 years living in Somaliland were identified with cirrhosis of otherwise unknown cause. All chewed khat habitually for many years (15–128 bundles per day times years of use). A liver biopsy of one man was consistent with khat hepatotoxicity. Four of the eight men died during the study period.

**Conclusion:**

Khat chewing may be associated with health consequences including severe hepatotoxicity with cirrhosis.

## Introduction

Chewing fresh khat (*Catha edulis*) is a common social custom in Somalia, Somaliland, Kenya, Ethiopia, Djibouti, and Yemen, especially among men. Cathinone and cathine are the active ingredients of khat. These compounds are structurally related to amphetamine. Users chew khat habitually for its euphoric effects. Most khat users today believe that khat is harmless.^1^ In contrast, most European Union member states and most G-7 countries have banned the exportation, importation, supply, possession and use of khat, because of khat’s potential for health and social harm^2,3^

There have been 12 case reports from the West^4,5,6,7,8,9,10,11,12,13,14,15^ that suggested an association between khat chewing and acute and chronic liver disease. The first report by D’Souza in 2005^4^ did not mention khat use, but a later study confirmed the association.^13^ These reports concerned 39 immigrants to the United Kingdom, United States, Holland, Belgium and Australia; 35 of the 39 cases were male, and 35 of 39 were ethnic Somalis.

Two reports regarding khat use originated from Djibouti, an area of indigenous use of regionally grown khat. Ardouin found no chronic liver disease in 204 liver biopsies among heavy khat users.^16^ Coton found no hepatitis among khat users between 2001 and 2007.^17^ Coton postulated that hepatotoxicity from khat was unique to immigrants to the West. It is not known whether toxicity might relate to the plant or to contaminants on the plant; nor is it known how chemical levels of the plant product in the blood relate to toxic effects. In addition, there are no other data about chronic liver disease in areas where khat is produced and used to determine if khat might in some cases be associated with cirrhosis. Thus, we reviewed findings of otherwise unexplained hepatic cirrhosis in Somaliland.

## Methods

Patient charts were reviewed to identify individuals with decompensated liver cirrhosis who presented to Al Hayatt Hospital in Borama, Somaliland, during 14 months beginning December 2012. All patients were interviewed to determine a possible cause for their cirrhosis. None of the patients had a past history of liver disease, blood transfusion or tattoos. All patients denied consumption of hepatotoxic drugs, herbal medicines and alcohol. None of the patients had indications of current or previous metabolic syndrome, making non-alcoholic fatty liver disease unlikely. Family history was negative for liver disease. There was no consanguinity among the parents or grandparents of the patients. Each patient underwent serologic testing for hepatitis B and hepatitis C. They were excluded from the study if they had evidence of hepatitis B or hepatitis C infection, alcoholic hepatitis or risk factors for fatty liver.

Borama is in a semi-arid area without year-round lakes and rivers where schistosomiasis is rarely identified. Resources are limited in this area, and patient care was provided consistent with local standards of care; expensive testing (such as serum albumin levels) and invasive evaluations (including liver biopsy) were only done when clinically indicated in this setting. The research was done retrospectively and did not impact the care of patients.

Admission history (including lifestyle habits such as the use of locally standard bundles of khat), physical exam, laboratory studies and patient progress were reviewed retrospectively. The research did not alter the clinical management of patients.

### Ethical considerations

This case series was reviewed and approved by the Amoud University Institutional Review Board.

## Results

As described in Table 1, there were eight cases identified; all were male. The average age of these patients at the time of presentation was 41 years. Seven of eight were subsequently seen in follow-up and had stopped chewing khat. One patient was lost to follow up despite efforts to find him. Three of the eight patients died from upper gastrointestinal haemorrhage within three months of presentation.

**TABLE 1:** Characteristics of patients with cirrhosis.

Patient	Age (yrs)	Khat use†	Presentation	Laboratory results‡				Ultrasound§	Quit khat	Outcome
				ALT	AST	ALKP	T Bili			
1	30	10	Ascites, spontaneous bacterial peritonitis, hepatic encephalopathy	118	50	352	2.8	Cirrhosis, splenomegaly	Yes	Died at home upper GI haemorrhage
2	33	22	Jaundice	20	15	522	5.5	Cirrhosis, splenomegaly	Yes	Improved without diuretics, after stopping khat
3	40	128	Hepatic encephalopathy, hepatorenal syndrome	54	42	310	2.7	Cirrhosis ascites	Yes	Died at home upper GI haemorrhage
4	27	15	Haematemesis, Jaundice, umbilical hernia	17	48	228	2.4	Cirrhosis, splenomegaly ascites	Yes	Improved with diuretics, after stopping khat
5	70	55	Haematemesis ascites	23	25	140	1.2	Cirrhosis	Unknown	Lost to follow up
6	30	60	Incarcerated umbilical hernia, ascites, spontaneous bacterial peritonitis	20	15	84	N.A.	Cirrhosis, splenomegaly ascites	Yes	Died in hospital upper GI haemorrhage
7	31	15	Jaundice, pneumonia,	64	100	80	11.5	Hepatomegaly, early cirrhosis	Yes	Improved
8	65	40	Ascites, itching	35	27	243	1.5	Cirrhosis ascites	Yes	Improved initially, then died at home. Cause?

*Source*: Authors’ own work

AST, is aspartate aminotransferase and is another name for SGOT (which is also called serum glutamic-oxaloacetic transaminase); ALT, is alanine aminotransferase and is another name for SGPT (which is also called serum glutamic-pyruvic transaminase); ALKP, alkaline phosphatase; T Bili, Total Bilirubin; GI, gastrointestinal; N.A, Study not done.

†, Bundle-years: bundles of khat chewed daily times the years of khat chewing. One bundle of khat weighs roughly 250 g.

‡, Normal range for ALT (SGOT): up to 48 u/L; AST (SGPT): up to 32 u/L; alkaline phosphatase: 40–306 u/L; total bilirubin: 0.1–1.0 mg/dl.

§, Criteria used for diagnosing cirrhosis by ultrasound: absence of capsular line, paucity of peripheral hepatic vessels, diminished echogenic wall of the portal vein, regeneration nodules with displacement of adjacent vessels, nodular liver contour, contracted liver, signs of portal hypertension.

**FIGURE 1:**
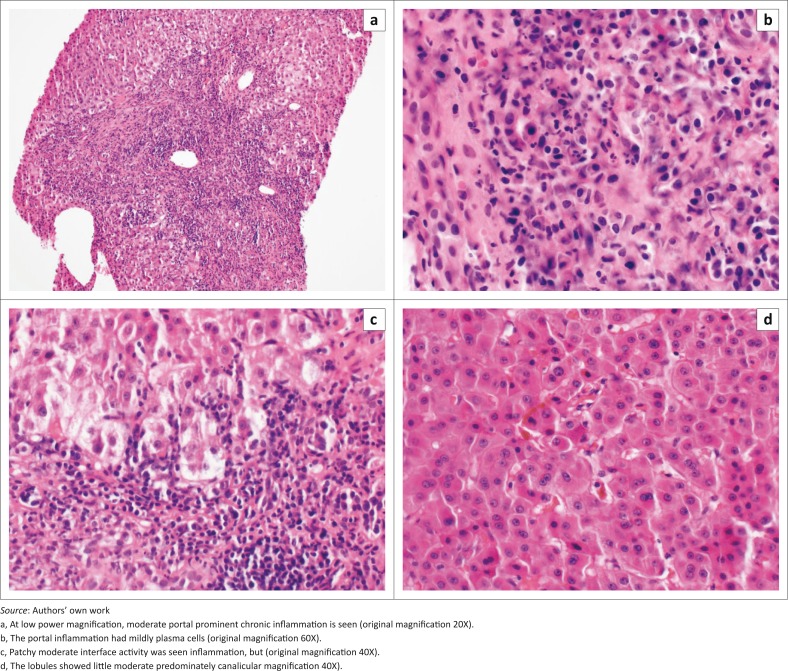
Liver biopsy.

A liver biopsy was done on patient number 3 in order to investigate the cause of cirrhosis. It showed changes of chronic hepatitis, with lobular cholestasis and advanced fibrosis. The pathology differential included autoimmune hepatitis, chronic viral hepatitis and drug effect. Histologically, there was moderate patchy portal inflammation (Figure 1a) that was composed predominately of lymphocytes but also had a mild prominence in plasma cells (Figure 1b) and occasional admixed eosinophils and neutrophils. There was focal mild bile ductular proliferation but no evidence for biliary obstruction. Interface activity was moderate but patchy (Figure 1c). The lobules show mild periportal hepatocyte swelling and moderate lobular cholestasis (Figure 1d). Inflammation in the lobules was mild and patchy. A single schistosome form was identified. The biopsy showed bridging fibrosis (METAVIR stage 3).

## Discussion

The cause of cirrhosis in these eight khat users is unknown. Common causes of cirrhosis, such as exposure to alcohol and hepatotoxic medications as well as viral infection, were excluded by history, physical exam and lab testing. Testing for the less common causes of cirrhosis was not available.

Khat toxicity is a plausible cause. All of the men in this case series reported daily consumption of khat for years. Animal research has shown that khat causes liver damage.^18^ Abid demonstrated a biochemical pathway for khat-induced liver damage on human liver cells.^19^ The studies cited above of Somali immigrants to Western countries showed that khat is associated with acute and chronic liver disease in humans, including autoimmune hepatitis. Four of our patients improved clinically after stopping khat consumption, and this improvement has been documented in other studies.^8,9,15^ Chapman demonstrated relapse of liver disease when consumption was resumed.^9^

The liver biopsy in one of our subjects is consistent with a drug effect. While this patient also had evidence of *Schistosoma* infection, the hepatic pathology was not consistent with changes typically seen with schistosomiasis-related portal hypertension. The observed histopathology is similar to results reported in other studies of khat and liver toxicity.^6,7,10,12,13^ Chapman showed that the histopathologic appearance of the liver of a patient who chewed khat and developed liver failure was similar to the changes caused by the recreational drug ecstasy, which, like khat, is related to amphetamine.^6^ Chapman also described a patient with acute liver failure who had a high concentration of cathinone in the liver 3 weeks after his last ingestion of khat, suggesting that drug accumulation in the liver might be toxic.^7^

It is possible that the liver damage could be caused, or aggravated, by pesticides or herbicides which are used in the cultivation of khat in Ethiopia, which is the source of most of the khat consumed in this part of Somaliland.^20^ Khat consumers in Yemen felt more ill after chewing khat that had been sprayed with DDT.^21^ In a cross-sectional study of 32 patients with Hirmi Valley Liver Disease (HVLD) in Ethiopia, Robinson showed that the probable cause of HVLD was the repeated ingestion of grain that contained acetyllycopsamine, a pyrrolizidine alkaloid that was found in the grain. DDT had been added to the stored grain, and DDT increases the toxicity of acetyllycopsamine.^22^ Cathine and cathinone are pyrrolizidine alkaloids.

There may be a genetic predisposition to hepatic injury from khat. Most reported cases involve Somali men. Robinson showed that the pathogenesis of HVLD is through the induction of cytochrome P450 by DDT.^22^ The induction of cytochrome P450 leads to the formation of toxic reactive acetyl free radicals that cause hepatocellular necrosis and cell death. Possibly, slow acetylators are more susceptible to khat hepatotoxicity than rapid acetylators.

## Conclusion

These cases suggest an association between khat chewing and liver toxicity, as previously proposed by studies involving mostly immigrant Somali men. These are the first case reports of possible khat hepatotoxicity that come from the region where khat is widely consumed, and from a country which is composed mostly of ethnic Somalis. Further research is needed to determine the relationship between khat and liver toxicity, its mechanism and natural history, the prevalence of liver toxicity in khat chewers in East Africa and Yemen, the influence of genetics on liver toxicity, and the role of pesticides and herbicides.

Many countries have banned khat because of its association with social and physical harm. But in the countries of East Africa and Yemen, where khat is produced and widely consumed, the public assumes it is harmless. If chewing khat is a hazard to health, then the people of these countries should be made aware of the risks.
